# The Impact of PCSK9 Inhibitors on Circulatory Inflammation: Mechanisms, Evidence, and Application Prospects

**DOI:** 10.1016/j.jacasi.2026.05.033

**Published:** 2026-08-04

**Authors:** Deng Bing-yan, Wang Jing-ya, Yuan Yu-juan, Dong Xiang-yu, Lai Hong-mei, Peng Hui, Yang Yi-ning, Yu Xiao-lin

**Affiliations:** a Graduate School of Xinjiang Medical University, Urumqi, China; b Department of Cardiology, People’s Hospital of Xinjiang Uygur Autonomous Region, Urumqi, China; c State Key Laboratory of Pathogenesis, Prevention and Treatment of High Incidence Diseases in Central Asia, Urumqi, China

**Keywords:** atherosclerosis, circulating inflammation, multimarker risk stratification, NLRP3 inflammasome, PCSK9, PCSK9 inhibitors

## Abstract

To systematically review and analyze the impact of proprotein convertase subtilisin/kexin type 9 (PCSK9) inhibitors on circulating inflammation, focusing on their underlying mechanisms and clinical application prospects, this review comprehensively summarizes recent studies addressing the molecular basis, nonclassical pathologic mechanisms, clinical evidence in multisystem diseases, therapeutic advances, and combined intervention strategies associated with PCSK9 and its inhibitors, highlighting their anti-inflammatory potential and personalized management strategies. PCSK9 participates in circulating inflammation primarily through multiple signaling pathways, including the NOD-like receptor family pyrin domain containing 3 inflammasome and the Toll-like receptor 4/nuclear factor kappa B axis. Clinical evidence indicates that PCSK9 inhibitors have limited direct effects on traditional inflammatory biomarkers. However, they show benefits in improving atherosclerotic plaque stability and modulating immune-inflammatory gene expression. The anti-inflammatory effects of PCSK9 inhibitors in circulating inflammation might not be directly reflected through changes in conventional inflammatory biomarkers. Future research should further explore their nonclassical mechanisms and optimize personalized risk stratification strategies, integrating multiomics and multiple biomarkers for precise inflammation management.

## Highlights

• Residual inflammatory risk persists despite intensive LDL-C lowering.
• PCSK9 inhibition improves plaque biology mainly via lipid-mediated effects.
• Conventional biomarkers show limited systemic anti-inflammatory effects.
• Future trials should standardize endpoints and refine subgroup risk.

Proprotein convertase subtilisin/kexin type 9 (PCSK9) has been a focus of research in the field of cardiovascular disease in recent years. It not only regulates cholesterol metabolism through the low-density lipoprotein receptor (LDLR) degradation pathway but is also closely related to the inflammatory response, immune regulation, and metabolic homeostasis.^1^ In brief, PCSK9 promotes lysosomal degradation of LDLR and increases circulating low-density lipoprotein cholesterol (LDL-C)/apolipoprotein B (apoB) lipoproteins, providing the canonical rationale for intensive lipid lowering with PCSK9 inhibitors.^1^ Because conventional systemic inflammatory biomarkers such as high-sensitivity C-reactive protein (hsCRP) often show minimal or inconsistent changes after PCSK9 inhibition, we interpret “inflammation-related” signals using a hierarchical framework that separates lipid-mediated plaque biological remodeling from direct systemic immunomodulation.^2^^,^^3^ Although traditional lipid-lowering therapy can significantly improve lipid profiles and reduce the risk for atherosclerotic cardiovascular disease (ASCVD) events, clinical practice shows that many patients still have considerable residual cardiovascular risk even after achieving lipid treatment goals, which is closely related to underlying chronic inflammation and immune disorders.^4^ Consistent with this view, a 2025 American College of Cardiology scientific statement reframes chronic, low-grade inflammation as clinically actionable across major cardiovascular syndromes and emphasizes the value of inflammatory risk assessment alongside lipid targets. This framework is particularly relevant for interpreting PCSK9 inhibitor trials in which plaque-level biological improvement may occur without a parallel, reliable reduction in conventional systemic biomarkers.^3^ Increasingly, basic and clinical evidence suggests that PCSK9 plays a crucial role in the circulating inflammatory network and immune regulation. In addition to the traditional lipid-lowering effect, PCSK9 inhibitors may also have potential benefits in anti-inflammation, immune regulation, and tissue protection.^5^^,^^6^

Therefore, it is of great theoretical significance and clinical value to closely analyze the regulatory mechanism of PCSK9 on circulating inflammation, systematically review the existing evidence related to PCSK9 inhibitors, and explore their future application prospects. To improve interpretability and avoid overextension of the term *anti-inflammatory*, we adopt a 2-level conceptual framework that distinguishes direct immunomodulatory effects from the lipid-mediated indirect anti-inflammatory impact of PCSK9 inhibition.^7^ Direct immunomodulation is defined as evidence that PCSK9 inhibition alters immune cell phenotypes or immune-inflammatory signaling in circulation (eg, immune cell functional properties), and such changes are not necessarily paralleled by reductions in conventional systemic biomarkers.^8^ In contrast, lipid-mediated indirect effects refer to downstream improvement in local plaque biology, such as reduced macrophage-rich plaque signals and more stable plaque phenotype, driven primarily by profound LDL-C lowering, which may occur without meaningful declines in hsCRP or broad cytokine panels.^9^^,^^10^ Accordingly, we interpret evidence hierarchically: large randomized controlled trial (RCT) biomarker data sets provide the strictest test for “systemic anti-inflammation,” whereas intracoronary imaging more directly informs local plaque inflammatory microenvironment but should not be equated with global inflammation suppression.^9^^,^^10^ This framework allows the mechanistic (often preclinical) literature to be integrated with human evidence while maintaining a clear boundary between mechanistic inference and clinically established anti-inflammatory effects.^1^ This review focuses on the molecular basis, classical function, nonclassical function, and pathogenic mechanism of PCSK9, as well as its clinical role and evidence-based evidence in circulatory diseases. It also examines drug development and combined intervention strategies, systematically summarizing and elaborating on the role and application prospects of PCSK9 inhibitors in regulating circulatory inflammation and multisystem diseases. Controversies in current research and future translational research directions are also discussed to provide a theoretical basis and a practical reference for the comprehensive prevention and control of cardiovascular diseases and related chronic diseases. Finally, given that Asian populations have different epidemiologic characteristics in baseline phenotypes, genetic polymorphisms, and medical access from Europe and the United States, in this paper we systematically integrate the evidence and guidelines for Asian populations and emphasize the regional specificity in the burden of intracranial atherosclerotic stenosis and stroke, compliance, and reimbursement patterns, to provide a basis for regional precision management.

## Molecular Basis and Classical Functions of PCSK9

PCSK9 is a secreted protein produced predominantly by the liver and released into the circulation, where it binds the LDLR and redirects the LDLR from recycling to lysosomal degradation.^1^ This LDLR-dependent pathway lowers hepatocyte surface LDLR abundance, thereby increasing circulating LDL-C/apoB-containing lipoproteins.^1^ Human genetics and pharmacologic inhibition collectively validate this canonical axis as the mechanistic foundation for the marked LDL-C reductions achieved by monoclonal antibodies and small interfering RNA (siRNA)–based PCSK9 inhibitors.^1^ As apoB lipoproteins are upstream drivers of plaque lipid accumulation and can amplify vascular wall inflammatory activation, intensive LDL-C lowering is expected to improve plaque burden and vulnerability.^9^ However, clinical data sets indicate that PCSK9 inhibition does not consistently reduce conventional systemic inflammatory biomarkers such as hsCRP, and meta-regression shows no significant correlation between changes in C-reactive protein (CRP) and LDL-C across lipid-lowering therapies.^2^ Therefore, in this review, the classical PCSK9-LDLR biology is intentionally summarized as background to support interpretation of predominantly lipid-mediated indirect effects rather than as evidence for established systemic anti-inflammatory efficacy.^3^ For detailed coverage of PCSK9 structure, trafficking, and genetic variation, readers are referred to comprehensive reviews.^1^

## Nonclassical Functions and Pathogenic Mechanisms of PCSK9

Mechanistic evidence linking PCSK9 to inflammation spans 2 conceptually distinct layers. First, direct immunomodulatory effects (lipid independent) are supported mainly by preclinical studies showing PCSK9-driven activation of innate immune pathways (eg, inflammasome-related signaling) or immune cell phenotypes even under LDLR-independent conditions.^11^^,^^12^ Second, indirect lipid-mediated effects are clinically robust: profound reductions in LDL-C and apoB-containing lipoproteins improve plaque burden and vulnerability features, but this does not necessarily translate into consistent reductions in conventional systemic inflammatory biomarkers such as hsCRP.^2^^,^^9^^,^^13^ Therefore, throughout this review, we explicitly distinguish mechanistic plausibility from clinically verified anti-inflammatory efficacy, and we interpret imaging readouts as local biological changes in plaque rather than as direct evidence of systemic inflammation suppression.

### Regulatory feedback mechanisms of inflammatory signaling

The role of PCSK9 in the regulation of inflammation has received increasing attention. Pcsk9 expression can be up-regulated by inflammatory stimuli such as TNF-α, IL-1β, LPS and oxidized low-density lipoprotein and is closely related to inflammatory markers such as CRP and IL-6 and chronic inflammation such as atherosclerosis and fatty liver.^5^ In hepatic HepG2 cells, TNF-α has been shown to increase PCSK9 expression through a SOCS3/STAT3-related mechanism, supporting cytokine-dependent regulation of PCSK9 under inflammatory conditions.^14^ PCSK9 can activate Toll-like receptor 4 (TLR4)/nuclear factor kappa B (NF-κB) and LOX-1 signaling pathways; promote the secretion of IL-1β, IL-18, MCP-1, and other proinflammatory factors; and drive the infiltration of macrophages and neutrophils to form a local chronic inflammatory microenvironment.^15^ In macrophage models, recombinant PCSK9 directly induces a proinflammatory phenotype, characterized by increased IL-1β, IL-6, TNF-α, CXCL2, and MCP-1 expression.^16^ There is a positive feedback regulation between PCSK9 and LOX-1, which further enhances oxidized low-density lipoprotein uptake and foam cell formation.^15^

Recent studies have shown that PCSK9 can promote the activation of NOD-like receptor family pyrin domain containing 3 (NLRP3) inflammasome and induce caspase-1-dependent IL-1β/IL-18 release and pyroptosis, which has been confirmed in diseases such as atherosclerosis, liver and myocardial injury.^17^^,^^18^ NLRP3/IL-1β signaling can, in turn, up-regulate PCSK9 through the MAPK pathway, and the 2 can form a positive feedback loop of metabolism and inflammation.^12^ PCSK9 can also cooperate with metabolic receptors such as CD36 and LRP5 to promote the formation of foam cells and the amplification of inflammatory signaling cascades.^19^

Although some large clinical trials have shown that PCSK9 inhibitors have a limited effect on traditional inflammatory markers such as CRP, the latest omics studies have suggested that PCSK9 inhibitors can reconstitute the expression of inflammatory genes in immune cells under a stressful environment, regulate NLRP3-pyroptosis and other pathways, and expand their anti-inflammatory effects to a new dimension.^8^

Collectively, available mechanistic studies support a bidirectional interplay between PCSK9 and inflammatory signaling, including an inflammasome-IL-1β axis that can regulate PCSK9 secretion^12^ and macrophage pathways linking lipid uptake to TLR4/NF-κB activation.^19^ Importantly, several key observations remain preclinical and should be interpreted as hypothesis supporting rather than clinically established immunomodulation. In humans, systemic inflammatory biomarkers often show limited or no incremental change despite intensive PCSK9 inhibition.^2^^,^^9^ These boundaries motivate a cautious translational framing: PCSK9 inhibition is a proven lipid-lowering strategy, whereas its direct anti-inflammatory efficacy in circulation requires dedicated clinical endpoints and validation.

### PCSK9-mediated cell death regulation and immune homeostasis imbalance

In recent years, the multiple functions of PCSK9 in programmed cell death and immune regulation have attracted significant attention, and it has become a crucial molecular link in multisystem pathologies such as inflammatory cardiovascular diseases, tumors, autoimmunity and metabolic abnormalities.^20^ PCSK9 not only affects cell death modes such as apoptosis, pyroptosis, ferroptosis, and autophagy but also profoundly shapes the immune microenvironment by regulating the function of T cell subsets, antigen presentation, dendritic cells, and B cells.^21^

A large amount of basic and clinical evidence has shown that PCSK9 can promote the apoptosis and pyroptosis of many types of cells, such as cardiomyocytes, vascular endothelial cells, and smooth muscle cells, and drive the progression of chronic inflammation, such as heart failure and atherosclerosis.^6^ Pcsk9 can enhance the sensitivity of myocardial and vascular cells to ischemia, oxidative stress and other stimuli by up-regulating proapoptotic factors such as Bax and caspase-3 and inhibiting antiapoptotic proteins such as Bcl-2 and promote structural degradation and dysfunction.^20^ In vascular smooth muscle cells, PCSK9 induces polyploidy, cell senescence, and apoptosis by down-regulating ApoER2, thus accelerating vascular ageing and vulnerable plaque formation.^22^

Pyroptosis, a type of programmed cell death that has attracted attention in recent years, is also prominent under the regulation of PCSK9. PCSK9 can promote mitochondrial DNA damage and reactive oxygen species accumulation, activate the NLRP3 inflammasome, drive caspase-1 and GSDMD cleavage, release IL-1β and IL-18, and eventually lead to pyroptosis of cardiomyocytes, endothelial cells, and other cells.^23^^,^^24^ Structural biology studies have shown that caspase-1/4/11 can cleave GSDMD with high affinity after self-cleaving, exposing its N-terminal domain, forming membrane holes and inducing pyroptosis.^25^ This mechanism provides a molecular basis for inflammatory cardiovascular disease. Inhibition of PCSK9 can down-regulate the expression of NLRP3/pyroptosis-related proteins and improve cell survival and cardiovascular outcomes.^6^^,^^26^

PCSK9 has also been implicated in ferroptosis-related redox imbalance and lipid peroxidation in cardiometabolic tissues, which may amplify inflammatory injury under ischemic or metabolic stress. Yet for circulating inflammation in ASCVD, the most interpretable human signals of PCSK9 inhibition currently come from 1) lipid lowering with downstream attenuation of plaque inflammatory biology; and 2) emerging studies showing that immune cell phenotypes may be unmasked under standardized ex vivo inflammatory stimulation (“stress” profiling) rather than from consistent reductions in conventional systemic biomarkers.^3^^,^^8^

In summary, PCSK9 has been implicated in regulated cell death programs that intersect with inflammatory amplification, including pyroptosis-related signaling in cardiomyocytes and endothelial cells in experimental models.^23^^,^^27^ Although these studies highlight mechanistic pathways through which PCSK9 inhibition could attenuate inflammatory cell injury, they rely largely on cell or animal systems and do not yet establish that PCSK9 inhibitors produce measurable systemic anti-inflammatory effects in patients as measured by conventional biomarkers. Accordingly, claims of “anti-inflammation” should be restricted to model-specific findings unless supported by human inflammatory endpoints. However, to avoid overgeneralization, the translational inferences of this review are anchored primarily in cardiovascular and metabolic contexts in which interventional human evidence for PCSK9 inhibition is available. Findings from noncardiovascular settings are therefore presented as emerging, hypothesis-generating signals rather than established clinical extensions.^3^^,^^6^

Box 1 **Noncardiovascular Immune Signals of PCSK9 Inhibition (Emerging, Hypothesis Generating)**

1. **Antigen presentation/MHC-I axis:** Experimental oncology studies suggest that PCSK9 inhibition may increase tumor MHC-I expression and enhance CD8^+^ T cell–dependent antitumor immunity; however, the relevance of this pathway to circulating inflammation control in ASCVD remains unproved. 2^28^
2. **Other organ system inflammation:** Reports in hepatic and immune-mediated conditions suggest potential PCSK9-inflammation crosstalk, but the evidence is predominantly preclinical and associative and should not be extrapolated as established “systemic anti-inflammatory” efficacy of PCSK9 inhibitors in humans.^3^

### PCSK9-related signaling network and multisystem homeostasis regulation

In recent years, PCSK9 has been increasingly viewed as a candidate node at the interface of lipid trafficking and immune-inflammatory signaling. In this review, we prioritize the cardiometabolic axis, in which PCSK9 inhibitors have robust interventional evidence, and interpret broader multisystem observations as context-setting biology rather than as proof of a universal clinical “hub” function.^3^^,^^5^^,^^8^^,^^15^

At the inflammatory signaling network level, PCSK9 can activate the TLR4/NF-κB pathway, enhance the expression of proinflammatory factors (IL-6, TNF-α, IL-1β), and cooperate with the LOX-1/oxidized low-density lipoprotein axis to promote macrophage activation, reactive oxygen species production, and foam cell formation, thereby driving atherosclerosis and chronic cardiovascular inflammation.^15^^,^^19^^,^^29^^,^^30^ In liver disease models such as nonalcoholic steatohepatitis and liver fibrosis, PCSK9 also participates in the process of hypoxia, inflammation, and fibrosis in liver tissue by regulating the AMPK/mTOR/ULK1 autophagy pathway and the NLRP3 inflammasome. PCSK9 inhibitors have shown effects on inhibiting the aforementioned signals and reducing inflammatory damage at both animal and cellular levels.^5^^,^^12^^,^^17^^,^^31^

Besides the LDLR, PCSK9 can synergize with CD36, a variety of receptors, such as LRP5, to enhance macrophage lipid intake, foam cell formation, and signal amplification of inflammation.^5^^,^^19^^,^^32^ More important, PCSK9 has an LDLR-independent effect on macrophage inflammation. Even in the absence of the LDLR, PCSK9 can still significantly promote the expression of inflammatory factors such as NLRP3/IL-1β by activating new signaling targets such as LOX-1 and syndecan-4. Even in the absence of LDLR, PCSK9 can still promote the expression of inflammatory mediators such as NLRP3 and IL-1β by activating emerging signaling targets such as LOX-1 and SDC4 (syndecan-4), thereby contributing to graft vascular inflammation and plaque instability and expanding the recognized role of PCSK9 in inflammatory regulation.^11^

Emerging structural and mechanistic studies suggest that PCSK9 can interact with MHC-I family proteins, such as HLA-C and HFE, as well as the adaptor protein CAP1, thereby regulating PCSK9-mediated lysosomal degradation of LDLR and potentially linking PCSK9 biology to antigen presentation, immune tolerance, and cellular immune function.^33^, ^34^, ^35^

The regulation of PCSK9 on the immune microenvironment is also worthy of attention. PCSK9 can not only down-regulate MHC-I expression, affecting the antigen presentation of dendritic cells and T cell function, but can also shape immune response and fibrosis by regulating micro-RNA, SIRT3, and other molecules.^5^^,^^36^ Multiomics analysis further demonstrates that PCSK9 plays a critical role in the chronic progression of various diseases across multiple systems, including the cardiovascular, hepatic, and cardiac systems, through the regulation of key biological pathways such as inflammatory responses, metabolic processes, and immune functions.^5^^,^^19^^,^^37^

In conclusion, as a multisignal hub of inflammation-metabolism-immunity, PCSK9 is involved in the progression of various chronic diseases, such as atherosclerosis, fatty liver, heart failure, immunometabolic diseases, and others. PCSK9 inhibitors not only reduce lipids but also partially reverse local and systemic inflammation and improve organ homeostasis through comprehensive intervention of multiple pathways, which has the triple potential of multidimensional anti-inflammation, immune remodeling, and metabolic intervention. In the future, the function and mechanism of its emerging signal axis (such as syndecan-4, CAP1/HLA-C complex, etc) should be further explored to provide theoretical bases and new strategies for the precise and comprehensive prevention and control of multisystem chronic diseases.^11^^,^^36^^,^^37^

## The Role and Clinical Evidence of PCSK9 in Circulatory Diseases

### The role of PCSK9 in cardiovascular diseases

PCSK9 is not only a key regulatory molecule of cholesterol metabolism but also plays an essential role in the occurrence and development of various cardiovascular diseases, such as atherosclerosis, heart failure, and stroke.^6^ The mechanism of PCSK9 is not limited to LDLR degradation and LDL-C elevation but also involves multiple links, such as vascular wall inflammation, foam cell formation, cell apoptosis, and energy metabolic remodelling.^6^^,^^38^ Basic and translational medicine research has revealed the multidimensional pathogenic mechanism of PCSK9 in the multisystem cardiovascular disease spectrum, which provides a theoretical basis for stratified management and targeted intervention of high-risk patients. Table 1 summarizes the mechanisms of action, key evidence, and clinical translational implications of PCSK9 in various cardiovascular diseases.

**Table 1:** PCSK9 in Major Cardiovascular Diseases

Types of Disease	Molecular Mechanisms and Pathways	Experimental and Clinical Evidence	Translational Implications and Commentary
Atherosclerosis/coronary heart disease	Promote LDLR degradation and increase LDL-C; aggravating vascular inflammation, foam cell formation, and plaque instability; activation of NLRP3 inflammasome and NF-κB pathway	High expression of PCSK9 promotes the progression of atherosclerosis and increases plaque vulnerability.^6^ Imaging evidence shows that plaque volume shrinkage and fibrous cap thickening are closely related to PCSK9 activity.^58^ Guidelines and reviews have confirmed that combination therapy is much better than a single incremental drug.^59^^,^^60^ NICE recommends specialist stratification and economic evaluation.^61^	Lipid management: For very high risk ASCVD, consider upfront combination therapy (high-intensity statin ± ezetimibe ± PCSK9i) to achieve ≥50% LDL-C reduction and LDL-C target of about <1.4 mmol/L, avoiding stepwise delay.^59^^,^^61^ Inflammation/residual inflammatory risk: The 2025 ACC scientific statement recommends hsCRP screening in both primary and secondary prevention and highlights “residual inflammatory risk” as clinically actionable. In this context, when PCSK9i produces robust LDL-C lowering but hsCRP is unchanged, “anti-inflammation” should be framed mainly as lipid-mediated plaque-level biological remodeling, and hsCRP can be used to identify patients who may need additional evidence-based anti-inflammatory strategies alongside LDL-C lowering.^3^
HF	Promotes pyroptosis, ferroptosis, lipid accumulation, and mitochondrial metabolism disorders in cardiomyocytes	Local PCSK9 deficiency in the myocardium leads to lipid accumulation and energy dysfunction.^38^ High expression of PCSK9 is associated with the progression of HF.^6^ The latest guidelines and consensus advocate multiomics and imaging monitoring.	Phenotyping and monitoring: Combine imaging, metabolomic phenotyping, and PCSK9-related risk markers to stratify HFpEF/HFrEF in high-risk patients (especially those with familial/functional variants).^6^ Inflammation framing: The ACC statement underscores the role of inflammation across CVD phenotypes, including HF. Inflammatory risk assessment (eg, hsCRP-based residual inflammatory risk) should be considered alongside LDL-C management when discussing “circulating inflammation” endpoints.^3^
Ischemic stroke	Can promote LDL-C elevation, aggravate vascular inflammation and endothelial injury, and enhance the nerve injury response	High expression of PCSK9 is associated with an increased risk for stroke.^4^ High PCSK9 expression in the acute phase predicts early deterioration.^62^ Guidelines recommend intensive intervention for high-risk populations of stroke.	Risk stratification: In high-risk stroke populations, integrate lipid risk and inflammatory risk profiling (hsCRP) rather than equating imaging/neurologic improvements with systemic inflammation suppression.^3^ Pragmatics: NICE-style frameworks emphasize access and cost-effectiveness when defining PCSK9i eligibility and sequencing.^61^
Peripheral vascular disease	Can affect arterial inflammation, regulate vascular smooth muscle cell migration/apoptosis, and promote disease progression	PCSK9-related gene variants are associated with risk for peripheral vascular events.^4^ Guidelines recommend combined lipid lowering in high-risk individuals.	Combined strategy: Early intensive lipid lowering for very high risk PAD patients, with inflammation risk assessment (hsCRP) considered in parallel when the paper specifically discusses “circulating inflammation” and residual risk.^3^ Implementation: family tracking and dynamic imaging/biochemical monitoring to individualize escalation; incorporate economic/access constraints as applicable.^61^

PCSK9 inhibition delivers consistent LDL-C lowering and plaque stabilization; per the 2025 ACC scientific statement, hsCRP-based residual inflammatory risk assessment should run in parallel, because systemic inflammatory biomarker reductions are not reliably captured despite local plaque-level biological improvement.

ACC = American College of Cardiology; ASCVD = atherosclerotic cardiovascular disease; CVD = cardiovascular disease; HF = heart failure; HFpEF = heart failure with preserved ejection fraction; HFrEF = heart failure with reduced ejection fraction; hsCRP = high-sensitivity C-reactive protein; LDL-C = low-density lipoprotein cholesterol; LDLR = low-density lipoprotein receptor; NF-κB = nuclear factor kappa B; NICE = National Institute for Health and Care Excellence; NLRP3 = NOD-like receptor family pyrin domain containing 3; PAD = peripheral artery disease; PCSK9 = proprotein convertase subtilisin/kexin type 9; PCSK9i = proprotein convertase subtilisin/kexin type 9 inhibitors.

Basic and clinical data have consistently confirmed that these multiple mechanisms of action of PCSK9 determine its unique position in the spectrum of cardiovascular multisystem diseases, which lays a theoretical and practical foundation for targeted PCSK9 intervention and precise stratified management of high-risk populations.^39^^,^^40^

### Multicenter RCTs, imaging, and long-term follow-up data

In the field of PCSK9 inhibitors, large multicenter RCTs, long-term follow-up studies, and imaging studies in the past decade have formed the evidence base for cardiovascular disease management practice. These studies have systematically evaluated multidimensional endpoints such as lipid-lowering durability, hard cardiovascular endpoints, plaque regression, and the safety of PCSK9 monoclonal antibodies (alirocumab, evolocumab) and siRNA drugs (inclisiran) in different high-risk populations. These studies have laid out the core position of PCSK9 in the precise management of ASCVD patients at very high risk. Overall, the evidence shows that PCSK9 inhibitors have clear benefits on hard endpoints such as major adverse cardiovascular events and all-cause death. Still, their effects on inflammatory markers show a contradiction between indirect anti-inflammatory effects and direct anti-inflammatory effects. On one hand, FOURIER (Further Cardiovascular Outcomes Research With PCSK9 Inhibition in Patients With Elevated Risk) and ODYSSEY Outcomes (Evaluation of Cardiovascular Outcomes After an Acute Coronary Syndrome During Treatment With Alirocumab) showed that PCSK9 inhibitors did not significantly reduce systemic inflammatory markers such as hsCRP, which supported that the anti-inflammatory benefits of PCSK9 inhibitors were based mainly on lipid-related mechanisms and plaque stabilization (ie, “traditional anti-inflammatory”).

However, imaging studies such as PACMAN-AMI (Vascular Effects of Alirocumab in Acute MI-Patients) and HUYGENS (High-Resolution Assessment of Coronary Plaques in a Global Evolocumab Randomized Study) have suggested that the fibrous cap of the plaque is thickening. In this review, imaging-reported “reductions in inflammation” (eg, decreased macrophage-related indexes and more stable plaque phenotypes on optical coherence tomography, intravascular ultrasound, and near-infrared spectroscopy) are interpreted as local plaque biological remodeling and should not be equated with systemic inflammation suppression in the absence of concordant changes in circulating biomarkers. The lipid core is shrinking, accompanied by a reduction in plaque inflammation and macrophage infiltration, indicating an improvement in the local inflammatory microenvironment. However, these changes are related primarily to the decrease in LDL-C. The evidence of direct anti-inflammatory mechanisms is limited.

It should be noted that some studies suggest that PCSK9 inhibitors may affect new inflammatory factors (such as IL-18, IL-6, sCD14, etc), but the vast majority of the results are not significantly different. The increasing trend of proinflammatory factors such as IL-18 was even observed in individual subgroups or analyses, suggesting the heterogeneity and complexity of direct anti-inflammatory effects. Tables 2 and 3 present the designs, endpoints, and inflammation-related evidence from representative studies.

**Table 2:** Inflammation Endpoints in Proprotein Convertase Subtilisin/Kexin Type 9 Studies: Large Outcome Trials and Long-Term Programs (Systemic Biomarkers as Endpoints or Safety Labs)

Study	Population	Intervention	Follow-Up	(Lipid/Clinical) Main Outcomes	(Inflammation-Systemic) Readouts Reported	Inflammation-Related Secondary Endpoints (if Any)
ODYSSEY Outcomes^63^^,^^64^	Post-ACS with prior MI; subgroup comparisons across TG levels	Alirocumab + statin	2.8 y	Reduced MACE; benefit consistent across lipid risk strata	NR (no prespecified circulating inflammatory panel in main report)	Not a prespecified inflammation-endpoint trial
FOURIER^65^	Stable ASCVD	Evolocumab + statin	2.2 y	Reduced CV events; robust LDL-C lowering	NR (no prespecified systemic inflammation endpoints in main report)	Not a prespecified inflammation-endpoint trial
FOURIER-OLE^48^^,^^66^	Extension of FOURIER	Evolocumab	Median ∼5 y	Long-term safety/efficacy extension	NR (inflammation biomarkers not a core endpoint)	Not a prespecified inflammation-endpoint trial
PACMAN-AMI^9^^,^^58^^,^^67^	AMI patients on high-intensity statins	Alirocumab + statin	52 wk	Strong LDL-C lowering; plaque phenotype/burden changes captured by intracoronary imaging	hsCRP: ↓ in both arms; between-group difference not significant	Imaging is the dominant biological readout (see Table 3)
ORION-8^42^	Open-label extension (long-term inclisiran exposure)	Inclisiran	4 y	Long-term LDL-C goal achievement and safety	NR (no prespecified circulating inflammation endpoints)	Not a prespecified inflammation-endpoint program
ORION-3^68^	Open-label extension of ORION-1	Inclisiran	4 y	Sustained LDL-C lowering; tolerability	NR (no prespecified systemic inflammatory biomarker panel)	Not a prespecified inflammation-endpoint program
ORION-5^69^	HoFH on maximal LLT	Inclisiran	150 d	LDL-C response heterogeneous (LDLR function dependent)	NR (no prespecified systemic inflammatory biomarker panel)	Not a prespecified inflammation-endpoint trial
ORION-10/ORION-11^70^	ASCVD/ASCVD risk equivalent	Inclisiran	510 d	Robust LDL-C lowering	NR (no prespecified systemic inflammation endpoints in primary reports)	Not a prespecified inflammation-endpoint trial

Across large outcome trials and long-term programs, proprotein convertase subtilisin/kexin type 9 inhibition delivers consistent lipid and clinical benefit, while systemic inflammatory biomarker evidence is limited or not reported, and imaging studies mainly capture local plaque biology. Per the latest American College of Cardiology framework, residual inflammatory risk should be evaluated explicitly (eg, hsCRP as a risk marker), and LDL-C lowering or plaque imaging improvement should not be overinterpreted as systemic anti-inflammatory efficacy.

ACS = acute coronary syndrome; AMI = acute myocardial infarction; CV = cardiovascular; FOURIER = Further Cardiovascular Outcomes Research With PCSK9 Inhibition in Patients With Elevated Risk; FOURIER-OLE = Further Cardiovascular Outcomes Research With PCSK9 Inhibition in Subjects With Elevated Risk Open Label Extension; HoFH = homozygous familial hypercholesterolemia; LLT = lipid-lowering therapy; MACE = major adverse cardiovascular events; MI = myocardial infarction; NR = not reported; PACMAN-AMI = Vascular Effects of Alirocumab in Acute MI-Patients; ODYSSEY Outcomes = Evaluation of Cardiovascular Outcomes After an Acute Coronary Syndrome During Treatment With Alirocumab; ORION-3 = An Extension Trial of Inclisiran in Participants With Cardiovascular Disease and High Cholesterol; ORION-5 = A Study of Inclisiran in Participants With Homozygous Familial Hypercholesterolemia (HoFH); ORION-8 = Trial to Assess the Effect of Long Term Dosing of Inclisiran in Subjects With High CV Risk and Elevated LDL-C; ORION-10 = Inclisiran for Participants With Atherosclerotic Cardiovascular Disease and Elevated Low-density Lipoprotein Cholesterol; ORION-11 = Inclisiran for Subjects With ASCVD or ASCVD-Risk Equivalents and Elevated Low-density Lipoprotein Cholesterol; TG = triglyceride; other abbreviations as in Table 1.

**Table 3:** Inflammation Endpoints in Proprotein Convertase Subtilisin/Kexin Type 9 Studies: Imaging Appendix (Local Plaque Biology Readouts; Do Not Equate With Systemic Inflammation Suppression)

Research	Design	Sample Size	Observation Period	Significant Imaging Findings (Local)	Systemic Inflammatory Markers	Investigators’ Preference for “Anti-Inflammatory/Stabilization”	Categorization
PACMAN-AMI^9^	Multicenter, double-blind, placebo-controlled RCT; IVUS + NIRS + OCT	300	52 wk	Greater PAV regression; maxLCBI_4mm decreased more; fibrous cap thickness increased; OCT macrophage angles/signals, etc, as local vulnerability readouts	There was no significant difference between hsCRP groups (*P* = 0.34)	The paper emphasizes “plaque regression/stabilization” and does not support systemic anti-inflammatory effects with hsCRP.	Biological improvement of local plaques (stabilization/regression)
HUYGENS^10^	Double-blind, placebo-controlled RCT; OCT + IVUS	161 (80 vs 81)	52 wk	Minimum fibrous cap thickness ↑; maximum lipid arc ↓; macrophage index ↓; PAV regression is greater	Unreported (at least not presented in abstracts)	The conclusion was worded as “consistent with stabilization and regression” and positioned as a “possible mechanism.”	Local stabilization phenotypes and systemic inflammation still require independent endpoint validation.
GLAGOV^71^	Placebo-controlled RCT; IVUS	968	76-78 wk	PAV regression (decreased in the evolocumab group, nearly zero change in the control group) demonstrated “enhanced lipid-lowering → plaque burden regression.”	Not reported (mainly baseline hsCRP inclusion factor in the text)	The main expression is “atheroma regression,” which is not equivalent to systemic anti-inflammatory.	Indirect lipid-mediated → local regression
ALTAIR^56^	Small sample RCT; OCT (TCFA vulnerability)	Unreported (PubMed does not provide an abstract)	Unreported	A decrease in vulnerability was indicated by indicators such as fiber cap thickness.	Not reported	The title “reduce vulnerability” leans more toward the “local stabilization” framework.	Local patch vulnerability improvement
FDG PET/CT arterial wall inflammation^13^	Multicenter, randomized, double-blind, placebo-controlled; PET/CT	129	16 wk	Arterial wall inflammation, MDSTBR, did not significantly decrease	CRP is not a good alternative to inflammation of the arterial wall, as discussed in the paper.	A clear distinction between “blood CRP” and “lesion inflammatory images”	Decoupling of local/systemic inflammatory endpoints
NIRS-IVUS short-term study^72^	Prospective single-arm, repeated measurements	27	2-6 wk	maxLCBI_4mm decreased significantly; decreased LCBI in the lesion (signal of very short term local lipid nucleus reduction)	Report changes in lipids and MDA-LDL, etc; systemic inflammatory markers were not reported	Emphasizing “plaque lipid nucleus reduction” rather than systemic anti-inflammatory	A rapid decrease in local lipid load

Across large outcome trials and long-term programs, proprotein convertase subtilisin/kexin type 9 inhibition delivers consistent lipid and clinical benefit, while systemic inflammatory biomarker evidence is limited or not reported, and imaging studies mainly capture local plaque biology. Per the latest American College of Cardiology framework, residual inflammatory risk should be evaluated explicitly (eg, hsCRP as a risk marker), and LDL-C lowering or plaque imaging improvement should not be overinterpreted as systemic anti-inflammatory efficacy.

ALTAIR = Alirocumab for Thin-Cap Fibroatheroma in Patients With Coronary Artery Disease Estimated by Optical Coherence Tomography; CRP = C-reactive protein; CT = computed tomography; FDG = fluorodeoxyglucose; GLAGOV = Global Assessment of Plaque Regression With a PCSK9 Antibody as Measured by Intravascular Ultrasound; HUYGENS = High-Resolution Assessment of Coronary Plaques in a Global Evolocumab Randomized Study; IVUS = intravascular ultrasound; LCBI = lipid core burden index; maxLCBI_4mm = maximum lipid core burden index within a 4-mm segment; MDA-LDL = malondialdehyde-modified low-density lipoprotein; MDSTBR = most diseased segment target-to-background ratio; NIRS = near-infrared spectroscopy; OCT = optical coherence tomography; PAV = percentage atheroma volume; PET = positron emission tomography; RCT = randomized controlled trial; TCFA = thin-cap fibroatheroma; other abbreviations as in Tables 1 and 2.

In addition, serial optical coherence tomographic, intravascular ultrasound, and near-infrared spectroscopic studies consistently demonstrate plaque regression and improvements in vulnerability features with PCSK9 inhibitors added to statins, including increased fibrous cap thickness and reduced lipid burden or macrophage-related signals.^9^^,^^10^ However, these imaging endpoints primarily reflect local plaque biology. In PACMAN-AMI, despite marked LDL-C lowering, hsCRP reduction did not differ significantly between treatment arms,^9^ and positron emission tomographic/computed tomographic data also suggest that arterial wall inflammation may remain unchanged in specific settings.^13^ Therefore, we describe these findings as plaque stabilization or regression rather than equating them with systemic inflammation suppression. Thus, in this paper we do not use negative hsCRP results to refute local plaque improvement, nor do we substitute imaging positivity for conclusions on systemic anti-inflammatory effects.

## Progress of PCSK9 Inhibitors

### Drug type (monoclonal antibody, siRNA, small molecule, gene editing, vaccine)

In recent years, PCSK9 inhibitors have shown a trend toward diversification in drug types and delivery strategies, from traditional monoclonal antibodies to new therapies such as siRNA, oral small molecules, peptides, nanovaccines, and gene editing, which have their own characteristics with respect to mechanism, lipid-lowering effect, compliance, and potential safety. Monoclonal antibodies, such as evolocumab and alirocumab, have established a robust evidence-based foundation in the management of adult and pediatric patients with heterozygous familial hypercholesterolemia.^41^ At the same time, siRNA-targeted interventions represented by inclisiran can significantly improve compliance with semiannual dosing.^42^ Oral small molecules, such as AZD0780 and MK-0616, have broken through the bottleneck of injection adherence.^43^^,^^44^ Vaccines,^45^ nanodelivery platforms,^46^ and clustered regularly interspaced short palindromic repeats–based editing^47^ represent the frontier for long-term and even potentially one-time cures.

There are significant differences in the mechanisms of action, lipid-lowering intensity, frequency of administration, long-term accessibility, and potential risks of different drugs. The relevant comparison is shown in Table 4, providing a reference for clinical individualized selection and multitarget combined intervention.

**Table 4:** PCSK9-Targeted Therapeutic Strategies

Types of Drugs (Main Literature)	Representative Drugs	Main Mechanism	LDL-C Reduction (Animal/Human)	Method of Administration	Frequency of Administration	Effect on Inflammation	Anti-Inflammatory Potential Rating	Key Advantages	Main Limitations
Monoclonal antibodies^13^^,^^41^^,^^73^	Evolocumab, alirocumab	Blocks PCSK9-LDLR binding and prolongs LDLR life	Human, ∼50%-60%	Subcutaneous injection	Every 2-4 wk	PET/CT showed reduced plaque inflammation. hsCRP did not decrease significantly.	★★★	There is sufficient evidence, MACE endpoint evidence, and broad indications.	Frequent injections are required, and the cost is high.
Fusion protein^74^	Lerodalcibep	The fusion protein prolongs half-life and blocks PCSK9.	Human, ∼55%-60%	Subcutaneous injection	Once a month	No inflammation endpoints; may inhibit the NLRP3 mechanism	★	The frequency of administration is low, and LDL-C is well controlled.	Inflammatory data are lacking, and there is no evidence of long-term safety and anti-inflammatory effects.
siRNA^42^^,^^50^^,^^75^	Inclisiran	Liver-targeted siRNA inhibits PCSK9 expression	Human, ∼45%-52%	Subcutaneous injection	Every 6 mo	RNA-seq suggested a remodeling of macrophage expression profile without a decrease in hsCRP.	★★	Drug compliance is high, liver targeting, and long-term effect	There were no significant changes in clinical inflammatory measures, and no imaging/endpoints were yet available.
Multiple targets of RNAi^76^^,^^77^	Olpasiran, ARO-ANG3	siRNA inhibits Lp(a) or ANGPTL3.	Lp(a) ↓ 80%-90%	Subcutaneous injection		Lp(a) promotes IL-6 and improves inflammatory phenotypes after intervention (animals).	★	Targeting residual risk, potent degradation	Direct inflammatory endpoints and plaque studies are lacking.
Oral small molecules/peptides^43^^,^^44^^,^^78^	MK-0616, AZD0780, etc	Macrocyclic peptides block PCSK9-LDLR.	Human, ∼50%-60%	Oral	Daily	No hsCRP or immune data, mechanism speculated	—	Noninjection and high compliance	Missing inflammation data
Epitope vaccines^73^^,^^79^	AT04A, AT06A	Epitope peptide plus adjuvant induces antibody neutralization of PCSK9.	Mice, ∼50%; plaque area ↓ 40%	Subcutaneous injection	Once or twice a year	The levels of SAA, MIP-1β, VEGF, and plaque inflammation were significantly decreased.	★★	Animal evidence is abundant and durable.	Clinical lipid lowering is limited, and inflammatory indicators are animal only.
VLP vaccine^80^	Bivalent VLP-PCSK9	VLP multiepitope antibody	Rhesus, LDL ↓ ∼25%-30%	Intramuscular injection	1 or 2 times/y	There were no inflammatory markers and no systemic inflammatory activation.	★	Good safety and durable immunity	There is a lack of inflammatory data, and clinical progress is early.
Ferritin NP vaccine^45^^,^^73^	HFn-PCSK9-NP	The ferritin vector expresses the PCSK9 segment.	Mouse/canine, LDL ↓ 50%-60%	Subcutaneous injection	Two immunizations	TNF-α/IL-12α was decreased, TGF-β was up-regulated, and foam cells/macrophages were decreased.	★★	The immunogenicity was stronger, and plaque inflammation was reduced.	Preclinical study with a small sample size
Nano-liposome vaccine^73^^,^^81^	L-IFPTA+	Epitope fusion + Th peptide, liposome delivery	Mouse, LDL ↓ 40%-50%	Subcutaneous injection	Once or twice a year	IL-10 was up-regulated, Th2 was polarized, and plaque inflammation was reduced.	★★	Adjustable immunophenotype, evidence of inflammation in animals	Not yet clinically advanced
Nano multivalent platform^46^	HFn-Pep2-8	Blocking PCSK9-LDLR at multiple sites	In vitro LDLR recovery ↑ 90%	On the hunt	Exploring	Foam cell formation/reduced inflammatory signaling (in vitro)	★	Multipathway inhibition with a flexible platform	Preclinical data, no in vivo inflammatory findings
Gene editing^47^	VERVE-101, ABE	Base editing to knock out PCSK9	Rhesus, PCSK9/LDL ↓ 60%	IV	Disposable	Decreased NLRP3/IL-1β inflammatory activity (animal)	★★	With potential one-time efficacy, the prospect is broad.	Off-target/ethical/inflammatory mechanisms need to be improved.

Current human evidence supports strong LDL-C lowering and plaque stabilization and regression, but systemic anti-inflammatory efficacy remains unproved or inconsistent; therefore, “anti-inflammation” should be reported as systemic biomarkers vs local plaque biology rather than a single interchangeable endpoint.

ANGPTL3 = angiopoietin-like protein 3; HFn = human ferritin; IL = interleukin; IV = intravenous; Lp(a) = lipoprotein(a); MIP-1β = macrophage inflammatory protein-1β; NP = nanoparticle; RNAi = RNA interference; RNA-seq = RNA sequencing; SAA = serum amyloid A; siRNA = small interfering RNA; TGF-β = transforming growth factor-β; Th = T helper; TNF-α = tumor necrosis factor-α; VEGF = vascular endothelial growth factor; VLP = virus-like particle; — = no clear anti-inflammatory evidence or mechanism support; ★ = only animal or mechanistic evidence support, or highly speculative; ★★ = supported by indirect human evidence or animal experiments and mechanism research; ★★★ = clear evidence from human studies (eg, decrease in inflammatory markers, radiographic reduction in inflammation); other abbreviations as in Table 1, Table 2, Table 3.

### Safety and compliance

PCSK9 inhibitors have shown significant advantages in drug safety and adherence. Monoclonal antibodies (eg, evolocumab, alirocumab) and siRNA drugs (eg, inclisiran) have been shown to be highly safe and well tolerated in large-scale RCTs and long-term follow-up. The most common adverse events were mild injection-site reactions, and serious adverse events were rare. There is no increased risk for new-onset diabetes, liver and kidney damage, or cognitive impairment in long-term very low LDL-C status.^41^^,^^48^^,^^49^ Emerging oral PCSK9 inhibitors, such as MK-0616, have further improved patient compliance and availability, with available data indicating a favorable safety profile.^43^^,^^44^ Semiannual injection can significantly improve long-term compliance among patients, especially for high-risk populations requiring lifelong management.^42^^,^^50^ These characteristics lay a solid foundation for the management and intervention of accurate risk stratification on the basis of multiple markers in the next step and provide a strong guarantee for the long-term individualized prevention and control of chronic cardiovascular diseases.

## Evidence in Asian Populations: Implications for Interpreting Circulating Inflammation and Implementing PCSK9 Inhibition

### The baseline phenotype and epidemiologic characteristics

In Asian cardiometabolic practice, intensive LDL-C lowering with PCSK9 inhibitors is frequently pursued on a background of predominantly moderate-intensity statin use, reflecting tolerability and prescribing patterns observed in regional trials.^51^ Meanwhile, cerebrovascular atherosclerosis phenotypes, in which plaque biology may be assessed by vessel wall imaging, are clinically prominent, and emerging Asia-based imaging data suggest that evolocumab add-on therapy can be associated with intracranial plaque regression.^52^ These practice-level features reinforce the central interpretive challenge of this review: local plaque remodeling can occur with profound LDL-C reduction, whereas conventional systemic inflammatory biomarkers should not be expected to decrease in parallel.^2^^,^^9^ Therefore, throughout the Asia-focused discussion, “inflammation-related benefit” is framed as either local plaque biological change or systemic biomarker change, and these 2 are not treated as interchangeable endpoints.^3^

### Genetics, drug response, and compliance

Genetic contexts relevant to lipid and inflammatory risk stratification, such as familial hypercholesterolemia driven by LDLR/APOB/PCSK9 variants, remain underdiagnosed and undertreated in parts of Asia, creating a sizable gap between baseline LDL-C levels and guideline targets and increasing reliance on combination lipid-lowering strategies.^51^ Regional development of locally available PCSK9 monoclonal antibodies (eg, tafolecimab) has expanded therapeutic options and may improve accessibility, but these trials primarily report lipid endpoints and safety rather than prespecified circulating inflammatory biomarker panels.^53^^,^^54^ Importantly, genetic or regional prescribing differences should not be used to infer established systemic anti-inflammatory efficacy. In line with the ACC framework, residual inflammatory risk should be evaluated explicitly (eg, hsCRP as a risk marker), and anti-inflammatory benefit should not be assumed from LDL-C lowering alone^3^^,^^55^ (Table 5).

**Table 5:** Asian Evidence for PCSK9 Inhibition

Study (Evidence Tier; Region; Therapy)	Population/Key Subgroups	Lipid Efficacy (Key Numbers)	Circulating Inflammation/Immune Readouts	Plaque/Local Biology Readouts	Key Asia Practice Note (PK/Dosing, Adherence, Safety; Interpretation Boundary)
FOURIER Asian subgroup (RCT subgroup analysis; across Asian sites; evolocumab)^82^	Stable ASCVD; Asian vs non-Asian comparison	LDL-C lowering was robust; LDL-C reduction was greater in Asians vs others (66% vs 58%)	NR (hsCRP/cytokines not prespecified in this subgroup report)	None	No excess serious AEs in Asians; no dose modification required based on race in this data set; “inflammation benefit” should not be inferred without circulating endpoints
ODYSSEY EAST (RCT; China/India/Thailand; alirocumab vs ezetimibe, 24 wk)^83^	High CV risk hypercholesterolemia on maximally tolerated statin	LDL-C −56.0% with alirocumab vs −20.3% with ezetimibe; 85.1% achieved LDL-C < 70 mg/dL	NR	None	Tolerability generally good; injection-site reactions low (2.7%); the lack of prespecified inflammatory readouts limits conclusions about direct systemic anti-inflammation
ORION-15 (RCT; Japan; inclisiran dose-ranging with PK)^84^	Hypercholesterolemia including HeFH; high CV risk	Highest dose (300 mg) achieved LDL-C −65.3% at day 180 (dose-response)	NR	None	Provides Asia-specific PK evidence supporting standard dosing; does not establish systemic anti-inflammatory effects because of missing circulating endpoints
ORION-18 (RCT; Asia; inclisiran vs placebo)^85^	ASCVD or high ASCVD risk; n = 345	LDL-C −57.2% at day 330; 71.7% achieved ≥50% LDL-C reduction	NR	None	Safety similar to placebo; inflammation claims should remain conservative because systemic inflammatory biomarkers were not a reported focus
CREDIT-4 (RCT; China; tafolecimab vs placebo, 12 wk)^86^	HeFH or high/very high CV risk; n = 303	LDL-C −68.9% vs −5.8% (placebo) at week 12; strong apoB and non-HDL-C reductions	NR	None	Example of regionally developed PCSK9 mAb expanding access; however, the absence of circulating inflammation endpoints prevents direct inference on systemic anti-inflammation
In-hospital ACS pilot study (case-crossover; China; evolocumab add-on)^87^	ACS patients during hospitalization (plus a non-CHD comparator cohort)	LDL-C decreased rapidly after a single dose of evolocumab (eg, median 109.0 mg/dL → 41.4 mg/dL at 72 h)	An exploratory cytokine panel suggested early changes, including decreases in IL-1β and IL-6 (not an outcomes-powered inflammation trial)	None	Represents an exploratory “circulating immune/cytokine” signal; it should be framed as hypothesis generating and not equivalent to validated systemic anti-inflammation
EPOCH (RCT; China; evolocumab + statin vs placebo + statin; 52 wk; CEUS)^88^	Premature CAD; newly diagnosed; n = 41	LDL-C percentage reduction −65% vs −32%; LDL target achievement 95% vs 53%	NR	Carotid IPN decreased more with evolocumab; imaging reflects plaque/local biology	Imaging benefit should be described as local plaque biology remodeling, not as systemic inflammation suppression.
Intracranial plaque regression study (comparative cohort; China; HR MRI)^52^	Statin-treated intracranial atherosclerotic stenosis ≥50%; n = 179 (50 evolocumab)	Evolocumab add-on associated with improved lipid profile parameters (HR MRI study context).	NR	Higher plaque regression response and greater plaque burden/stenosis reduction with evolocumab add-on (HR MRI)	Supports local plaque improvement in an Asia-relevant phenotype; an inflammatory interpretation should remain local/plaque-centered unless circulating biomarkers are concordant
ALTAIR AI-aided OCT analysis (randomized study; Japan; alirocumab + statin vs statin; 36 wk)^89^	Thin-cap fibroatheroma; n = 24	LDL-C lowering expected with add-on PCSK9i (study focuses on plaque phenotype)	NR	Greater fibrous cap thickening and lipid component reduction by AI-aided OCT; macrophage-related signals are local readouts	Use wording such as “plaque stabilization/regression phenotype”; avoid equating OCT plaque signals with systemic inflammation suppression.
Japan postmarketing real-world study (single arm; Japan; evolocumab; 104 wk)^90^	HoFH/HeFH and high-risk hypercholesterolemia; safety set n = 3,724	LDL-C reductions at week 12: HoFH, −45.7%; HeFH, −55.9%; high risk, −63.3% (mean ± SD)	NR	None	Reinforces feasibility and tolerability in Japan; does not provide circulating inflammation endpoints
Korea PMS (prospective PMS; Korea; evolocumab; up to 52 wk)^91^	ASCVD or FH; safety set n = 539	LDL-C reduced from baseline median 100.2 mg/dL by 70.6% at week 12 and 69.0% at week 52	NR	None	Real-world safety is favorable across achieved LDL-C strata; the study supports lipid management but cannot determine the effect on systemic inflammation.

In Asian-representative studies, PCSK9i consistently deliver profound LDL-C lowering with reasonable safety and implementation feasibility. However, most trials and real-world programs do not prespecify, or report circulating inflammatory biomarkers, and imaging improvements should be interpreted as local plaque biological remodeling rather than as proven systemic inflammation suppression.

AE = adverse event; AI = artificial intelligence; apoB = apolipoprotein B; CAD = coronary artery disease; CEUS = contrast-enhanced ultrasound; CHD = coronary heart disease; CREDIT-4 = A Study of IBI306 in Participants With Hypercholesterolemia; EPOCH = Effect of Combining Evolocumab With Statin on Carotid Intraplaque Neovascularization in Patients With Premature Coronary Artery Disease; FH = familial hypercholesterolemia; HDL-C = high-density lipoprotein cholesterol; HeFH = heterozygous familial hypercholesterolemia; HR = high-resolution; IPN = intraplaque neovascularization; mAb = monoclonal antibody; MRI = magnetic resonance imaging; ODYSSEY EAST = Evaluation of Alirocumab Versus Ezetimibe on Top of Statin in Asia in High Cardiovascular Risk Patients With Hypercholesterolemia; ORION-15 = Study of Efficacy and Safety of Inclisiran in Japanese Participants With High Cardiovascular Risk and Elevated LDL-C; ORION-18 = Study of Efficacy and Safety of Inclisiran in Asian Participants With Atherosclerotic Cardiovascular Disease (ASCVD) or ASCVD High Risk and Elevated Low Density Lipoprotein Cholesterol; PK = pharmacokinetic; PMS = postmarketing surveillance; other abbreviations as in Tables 1 to 3.

### Clinical evidence and imaging subgroups in the Asian population

Large-scale randomized evidence demonstrates that PCSK9 inhibition reduces major cardiovascular events, and this overall benefit is consistent across primary clinical settings; thus, we do not restate global efficacy and safety results in detail here.^4^ Instead, we emphasize the inflammation-specific evidence gap in Asian-representative studies: conventional systemic biomarkers (hsCRP and broad cytokine panels) are often not prespecified or show limited incremental change, consistent with comprehensive meta-analytical evidence indicating no meaningful correlation between CRP change and LDL-C change across lipid-lowering therapies and suggesting no consistent hsCRP reduction with PCSK9 inhibitors.^2^ In contrast, imaging studies, including Asian optical coherence tomography data demonstrating fibrous cap thickening and reduced macrophage-related plaque signals, support plaque stabilization and regression phenotypes.^56^ However, these imaging endpoints primarily reflect local plaque biology and should not be equated with systemic inflammation suppression, particularly when systemic biomarker changes are absent or unreported.^9^^,^^10^^,^^13^

### Guide practice and accessibility

For Asian practice, we propose an inflammation-informed implementation pathway that treats residual cholesterol risk and residual inflammatory risk as parallel but distinct domains. The 2025 American College of Cardiology scientific statement highlights that chronic low-grade inflammation is clinically actionable and provides practical recommendations for inflammatory risk assessment alongside lipid targets.^3^ Accordingly, PCSK9 inhibitors should be positioned primarily as intensive lipid-lowering therapies to address residual cholesterol risk, whereas claims of “systemic anti-inflammation” should be supported by concordant circulating biomarker endpoints rather than inferred from imaging alone.^2^^,^^9^ Access, reimbursement, and dosing convenience materially influence the feasibility of sustained risk factor control; Chinese cost-utility modeling suggests that negotiated pricing is a key determinant of cost-effectiveness, underscoring the need for region-specific pathway design.^53^ Continued regional development of PCSK9-targeting agents may improve accessibility, enabling more consistent LDL-C control and thus supporting plaque-level biological improvement, while inflammation management should be guided by validated biomarkers and dedicated trials.^3^^,^^54^

## Future Prospects and Conclusions

In recent years, PCSK9 inhibitors have become an essential tool for the prevention and treatment of cardiovascular diseases because of their excellent lipid-lowering efficacy and good safety. A large number of studies have shown that PCSK9 not only regulates cholesterol metabolism but also is deeply involved in circulating inflammation, programmed cell death, and immune homeostasis, which provides a theoretical basis for its potential anti-inflammatory effect. However, clinical studies have shown that the impact of PCSK9 inhibitors on traditional inflammatory markers (CRP and IL-6) is still controversial, and the anti-inflammatory benefits of PCSK9 inhibitors are more reflected in plaque stabilization and improvement of cardiovascular endpoints. In line with the conceptual framework proposed in the introduction, current human evidence more consistently supports lipid-mediated indirect effects, manifesting as local plaque biological remodeling and improved plaque phenotype. In contrast, evidence for direct, systemic immunomodulation remains limited or inconsistent when judged by circulating biomarkers. As summarized in the Central Illustration, PCSK9 is linked to inflammation-related biology through multiple lipid, immune, and cell-stress pathways; however, mechanistic plausibility should not be equated with uniform clinical evidence for direct systemic anti-inflammatory effects. Accordingly, imaging-reported “inflammation reduction” should be interpreted as changes in the local plaque inflammatory microenvironment and not automatically equated with systemic inflammation suppression in the absence of concordant biomarker shifts. This dissociation suggests that clinically observed benefits may extend beyond changes captured by conventional circulating biomarkers and may involve plaque-level biologic remodeling or other context-dependent pathways. In some patients, the traditional inflammatory markers may not change significantly because of the redundancy and alternative signals of inflammatory pathways, but they can still achieve clinical benefits. This hypothesis warrants further exploration. Notably, diabetes mellitus represents a high-risk immunometabolic phenotype characterized by chronic low-grade inflammation; therefore, future PCSK9 inhibitor studies should explicitly examine whether inflammatory endpoints and plaque-level responses differ in diabetes and other high-inflammation subgroups.^3^^,^^57^ To rigorously test direct anti-inflammatory effects, future trials should prespecify inflammation-focused endpoints, adopt standardized and clinically accessible assays (eg, hsCRP and key cytokine pathways), and implement multimarker stratification frameworks with harmonized sampling and analysis pipelines, as highlighted in contemporary consensus on inflammation and cardiovascular disease.^3^ The 2025 American College of Cardiology scientific statement provides a practical framework for interpreting “inflammation” in cardiovascular medicine, emphasizing risk assessment and careful interpretation of biomarkers and imaging signals. Within this framework, the most defensible reading of current evidence is that any inflammation-related benefits observed with PCSK9 inhibitors are predominantly indirect, driven by profound apoB and LDL-C lowering and downstream plaque biological stabilization, rather than a consistent suppression of circulating inflammatory biomarkers.^3^ Direct systemic anti-inflammatory effects remain uncertain and are likely underestimated by conventional endpoints alone; future trials should therefore prespecify inflammation-relevant outcomes beyond hsCRP, including standardized immune cell functional profiling under controlled inflammatory stimulation and multimarker and omics-based stratification.^3^^,^^8^

**Central Illustration:**
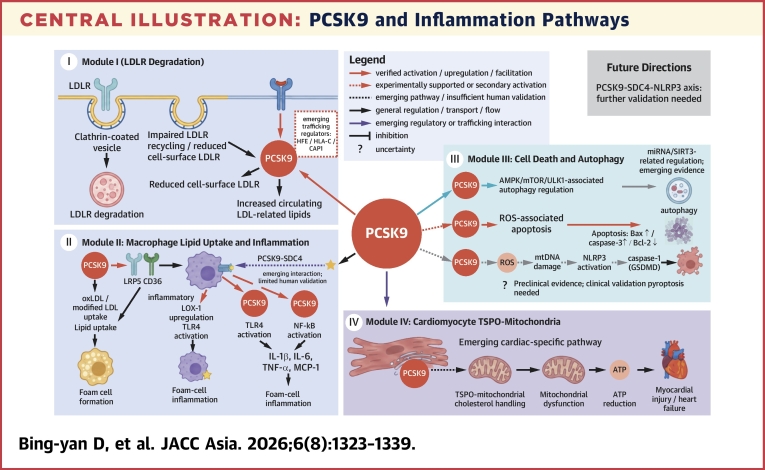
PCSK9 and Inflammation Pathways Proprotein convertase subtilisin/kexin type 9 (PCSK9) promotes low-density lipoprotein receptor (LDLR) degradation and increases circulating low-density lipoprotein (LDL)–related lipids. In macrophages, PCSK9 enhances lipid uptake and inflammatory activation through LDL receptor-related protein 5 (LRP5)/cluster of differentiation 36 (CD36), lectin-like oxidized LDL receptor-1 (LOX-1), Toll-like receptor 4 (TLR4)/nuclear factor kappa B (NF-κB), and emerging syndecan-4 (SDC4)–related interactions. PCSK9 is also linked to reactive oxygen species (ROS)–associated apoptosis, preclinical NOD-like receptor family pyrin domain containing 3 (NLRP3)/caspase-1/gasdermin D (GSDMD)–mediated pyroptosis, and AMP-activated protein kinase (AMPK)/mechanistic target of rapamycin (mTOR)/unc-51-like autophagy activating kinase 1 (ULK1)–associated autophagy regulation. Emerging cardiac-specific evidence suggests that PCSK9-related translocator protein (TSPO)–mitochondrial cholesterol handling may contribute to mitochondrial dysfunction, adenosine triphosphate (ATP) reduction, and myocardial injury or heart failure. CAP1 = cyclase-associated protein 1; HFE = homeostatic iron regulator; HLA-C = human leukocyte antigen C; IL = interleukin; MCP-1 = monocyte chemoattractant protein-1; miRNA = micro-RNA; mtDNA = mitochondrial DNA; oxLDL = oxidized low-density lipoprotein; SIRT3 = sirtuin-3; TNF-α = tumor necrosis factor-α.

Future research should prioritize the following directions:

1. Determine the direct anti-inflammatory targets of PCSK9 inhibitors, such as blocking PCSK9-CD36 interaction and inhibiting the NLRP3 pathway.
2. Explore special groups, such as heart failure, chronic kidney disease, and diabetes mellitus, and the differences in PCSK9 inhibitors’ inflammatory response, and clarify individual response mechanism and residual inflammatory risk patterns.
3. Clarify the context-specific role of PCSK9 in acute coronary syndromes and the post-acute inflammatory milieu, and determine whether PCSK9 inhibition is associated with changes in immune-cell activation, residual vascular inflammation, or plaque-level inflammatory biology beyond LDL-C lowering.
4. Further elucidate the mechanism of PCSK9 on monocyte and macrophage phenotyping, especially on the migration, differentiation, and chemokine expression of inflammatory cells, aiming to reveal a new dimension of its anti-inflammatory mechanism.
5. Conduct Asian population–oriented implementation and cost-effectiveness studies to optimize region-specific use of PCSK9-targeting therapies, considering stroke and intracranial atherosclerosis burden, PCSK9/LDLR genetic variation, baseline LDL-C profiles, reimbursement systems, and long-term adherence.

In addition, the clinical translation of PCSK9 inhibitors also faces significant challenges:

1. The cost of innovative therapies, such as gene editing and vaccine therapy, is high, and economic accessibility must be addressed.
2. The clinical standardization and accessibility of inflammatory biomarkers, such as IL-1β detection methods, must be improved.
3. Large-scale studies are needed to verify the practicality of multimarker combined stratification and to update guidelines.
4. The long-term safety and specific indications of new therapies in different populations must be continuously monitored and improved.

These translational challenges are significant for differentiating direct systemic immunomodulation from lipid-mediated plaque-level changes and for identifying subgroups (eg, diabetes) in which residual inflammatory risk may persist despite optimal LDL-C lowering.^3^

In summary, PCSK9 inhibitors have the potential for multitarget intervention in metabolism, inflammation, and immune regulation. In the future, it is necessary to focus on clarifying the direct anti-inflammatory mechanism of PCSK9 inhibitors, paying attention to differences in response among special populations, and solving the problems of the economy of innovative therapies and standardization of marker detection. Through multidisciplinary collaboration and long-term follow-up studies, accurate and individualized management of circulating inflammation is expected to be truly realized, which will provide a solid foundation for the comprehensive prevention and control of cardiovascular diseases and related chronic diseases.

## Funding Support and Author Disclosures

This work was supported by the Natural Science Foundation of Xinjiang Uygur Autonomous Region (grant 2023D01091) and the Tianshan Talent Cultivation Program-High-Level Medical and Health Professionals Project, Xinjiang Uygur Autonomous Region (grant TSYC202301B006). The authors have reported that they have no relationships relevant to the contents of this paper to disclose.
